# Efficacy of Chitosan-Carboxylic Acid Hydrogels in Reducing and Chelating Iron for the Removal of Rust from Stone Surface

**DOI:** 10.3390/gels10060359

**Published:** 2024-05-22

**Authors:** Francesco Gabriele, Cinzia Casieri, Nicoletta Spreti

**Affiliations:** Department of Physical and Chemical Sciences, University of L’Aquila, 67100 L’Aquila, Italy; cinzia.casieri@aquila.infn.it (C.C.); nicoletta.spreti@univaq.it (N.S.)

**Keywords:** iron rust, lithic surface, cleaning, chitosan hydrogel, reducing agent, chelating agent

## Abstract

In the field of stone conservation, the removal of iron stains is one of the most challenging issues due to the stability and low solubility of the ferrous species. In the present paper, three different chitosan-based hydrogels added with acetic, oxalic or citric acids are applied on different lithotypes, i.e., granite, travertine and marble, widely diffused in monumental heritages, and artificially stained by deposition of a rust dispersion. The reducing power of carboxylic acids is combined with the good chelating properties of chitosan to effectively remove rust from stone surfaces. As evidenced by colorimetry on three samples of each lithotype and confirmed by ^1^H-NMR relaxometry and SEM/EDS analyses, the chitosan-oxalic acid hydrogel shows the best performance and a single application of 24 h is enough to get a good restoration of the stone original features. Lastly, the chitosan-oxalic acid hydrogel performs well when a rusted iron grid is placed directly on the lithic surfaces to simulate a more realistic pollution. Current work in progress is devoted to finding better formulations for marble, which is the most challenging to clean or, with a different approach, to developing protective agents to prevent rust deposition.

## 1. Introduction

Removal of rust stains from stone materials, especially in the field of monumental heritage, is one of the most challenging cleaning actions due to the high thermodynamic stability of iron oxyhydroxides and hydrated oxides. Iron is often in contact with stone materials in cultural heritages; in fact, iron bars, nails, and other decorative or support objects are diffused elements in lithic works of art [1,2,3]. Moreover, iron can be found in minerals of lithic substrates, such as in the form of pyrite (FeS_2_), siderite (FeCO_3_) or biotite (K(Mg,Fe^2+^)_3_AlSi_3_O_10_(OH,F)_2_) and others [4]. Their continuous exposure to pollutants, particulate, acid rain, and humidity induces the formation of numerous iron oxidation products and the appearance of yellow-brownish and red-blackish stains on the stone substrate [5]. The presence of rust products not only affects the aesthetics of the artwork but also leads to degrading actions of the lithic supports and even to the formation of cracks. This phenomenon is caused by the “rust expansion” effect for which the oxidized metal undergoes an increase in its volume over time [6,7].

Water plays an important role for iron oxyhydroxide nanoparticles, both as a dispersing medium and as a carrier through the stone. Then, the nanoparticles can interact with the substrate and undergo a series of transformations before being converted into more stable iron compounds [8,9]. There is a plethora of iron-based compounds that form rust, which can interconvert each other according to the environmental conditions, i.e., pH, relative humidity (RH), and the counterions involved in the oxidation processes. In a dried oxygen-poor environment, a mixed ferrous/ferric oxide is mainly formed, and the so-called magnetite (Fe_3_O_4_) appears as a dark stain. In contrast, under high RH conditions, the iron-hydrated oxides and hydroxides are predominant.

The thermodynamic constants and the solubility of some of the main rust constituents, such as ferrihydrite, α-, β- and γ-FeOOH, were investigated and goethite (α-FeOOH) was found to be the most stable and insoluble (solubility product constant, K_sp_ = 10^−41^) [5,10,11]. Therefore, being the most thermodynamically stable, an effective cleaning of rust stains must ensure the complete removal of goethite from the lithic substrate [5].

Nowadays, physical or chemical approaches are used to remove iron stains from stone surfaces, also in the field of cultural heritage. The first ones are generally mechanical methods, and recently, good results have been reached on granite by using laser cleaning. This technique reduces the typical loss of substrate layers due to the abrasive blasting and ensures a safer removal of rust products [12].

Chemical approaches, the most diffused in conservation practice, involve the use of chelating or reducing agents. Aqueous solutions of ammonium citrate or ethylenediaminetetraacetic acid (EDTA) are applied to bind iron and remove it from artworks [13]; differently, reducing agents, such as oxalic acid and sodium dithionite or thioglycolic acid are employed to increase the solubility of iron and allow its removal from the stone [14,15] or from old iron objects [16], respectively. However, chemical treatments often lead to the deposition of unwanted residues even if the substrate surface is rinsed with water, as well as to the removal of calcium ions from carbonate substrates. Poultices of cellulose, carboxymethyl cellulose (CMC), or other materials, commonly used to clean stone surfaces [17,18], can incorporate reducing and chelating agents to effectively remove iron oxyhydroxides and by-products from the artwork surface [3,5]. Nevertheless, by adopting this procedure on carbonate stones, the calcium ions of the substrate could also be sequestered, thus damaging the surface of the stone [3]. By comparing the side effects of the two chelating agents on marble under the same experimental condition, ammonium citrate turns out less aggressive than EDTA, albeit only partially removing the calcium ions [19]. A recent study has shown a new green methodology to effectively chelate and remove Fe (III) on artificially stained marble samples by using proteins, such as ovotransferrin and lactotransferrin [20]. Due to the high specificity of these proteins for ferric cations, they should not interact with the substrate, including carbonates, to ensure the integrity of the material. Oxalic acid used as a reducing agent proved to be a good strategy to effectively remove rust stains from sandstone when applied together with doped cellulose poultice [14]. In this way, Fe(III) species reduced to Fe(II) compounds are generally more soluble, i.e., Fe(OH)_2_ shows a K_sp_ of about 10^−14^ and therefore could easily diffuse into the poultice support. More recently, a viscous DES composed of oxalic acid and choline chloride has shown high efficacy in removing iron oxides from both cellulosic and lithic-stained substrates [21].

To achieve a more effective rust removal, the treatment often involves the combined use of reducing and complexing agents. In fact, in the last two decades, several works have been aimed at finding the optimal pH and the more effective combination of reducing and chelating agents to successfully remove iron stains without compromising the integrity of the stone substrates. Sodium dithionite seems to be a good reducing agent that can easily reduce Fe(III) to Fe(II) and then can be coupled with different chelating agents to remove iron from stone substrates. Among the common ligands, cysteine forms strong complexes with Fe(II) that could be easily transferred into suitable cellulose poultice [5,22]. However, the optimum working pH of this last system is less than 10, which represents the threshold value for the partial dissolution of the calcium carbonate. To avoid this problem, cysteine can be replaced with hexadentate N,N,N′,N′-tetrakis(2-pyridylmethyl)ethylenediamine (TPEN) that works efficiently even at a pH of about 10. Unlike EDTA, this chelating agent is highly selective towards heavy metal ions over calcium ones, making it less aggressive to carbonate stone substrates [23]. Another alternative is the biodegradable tetrasodium 3-hydroxy-2,2′-iminodisuccinate (HIDS), which has been shown to be effective in removing rust stains from cotton fabrics when combined with sodium dithionite as a reducing agent [24].

Although most reported investigations describe the use of pure or doped cellulose poultice as a medium to convey cleaning agents for the removal of pollutant species, the application of nanostructured fluids or hydrogels has increased significantly in recent years [25,26,27]. For example, polysaccharide-based hydrogels encapsulating biocides of different natures have been effectively employed to remove microbial colonizers from biodeteriorated stone surfaces, both in the laboratory and in situ [28,29,30]. The use of agar gels in the cleaning procedures for various conservation interventions has been summarized by Sansonetti and coworkers [31]. Inter alia, the review counts on the efficacy of agar-chelating agent gels in removing copper stains from marble surfaces [32]. Moreover, agar hydrogels, incorporating ionic liquids, have been applied to sequester iron and copper ions from stone materials, and the effectiveness of the formulations, selected by laboratory tests, have been validated in situ on naturally stained substrates [33]. Recently, an EDTA-loaded bacterial nanocellulose hydrogel has been able to completely remove copper stains from marble, proving to be an effective alternative to traditional hydrogels for the cleaning of cultural heritage materials [34].

In this paper, chitosan-based hydrogels were prepared for the removal of stable rust products from the surface of different stone lithotypes. Chitosan is a linear polysaccharide that derives from the partial deacetylation of chitin; it is composed of β (1-4)-linked d-glucosamine and N-acetyl-d-glucosamine disposed randomly along the chains [35]. It is soluble only in weakly acid-aqueous solutions (pK_a_ ≈ 6.5) and, at high polysaccharide concentrations, its chains can become entangled, leading to the formation of a physical hydrogel [36,37]. Its high biocompatibility, biodegradability, and intrinsic bacteriostatic efficacy also show good chelating properties. In fact, thanks to the large number of hydroxyl groups, the active ammino groups, and the flexible polymeric structure, chitosan is generally suitable for the adsorption of heavy metal ions. To enhance the complexing ability of the hydrogel in the removal of silver from aqueous solutions, promising results have been obtained using chemically crosslinked chitosan encapsulating a siderophore [38]. In addition, physical chitosan hydrogel dissolved in an acid solution and added with thiourea dioxide as a reducing agent has been effective in removing manganese stains from both glass and marble [39].

Despite the good chelating properties of both pure, modified and crosslinked chitosan towards Fe(II) and Fe(III) [40,41,42], to the best of our knowledge, there are no papers dealing with the use of chitosan-based hydrogels for the removal of iron oxides from stone surfaces. For this purpose, acetic, oxalic, and citric acids have been selected for reducing insoluble Fe(III) species to more soluble Fe(II) ones and, at the same time, to dissolve chitosan, which was selected as a chelating agent capable of forming three different hydrogels: chitosan-acetic acid (CS-Ac), chitosan-oxalic acid (CS-Ox) and chitosan-citric acid (CS-Cit).

Three lithotypes, i.e., granite, travertine, and marble, widely diffused in-built heritages and characterized by different compositions and porosity, have been artificially stained and treated with each of the three chitosan-based hydrogels. Firstly, colorimetry has been used to select the best formulation on a macroscopic scale. Then, ^1^H-NMR relaxometry, SEM-EDS analysis, or stereomicroscope observation was performed to evaluate the effectiveness of the selected hydrogel system.

## 2. Results and Discussion

The viscosity of the reducing-chelating hydrogels, composed of chitosan and acetic, oxalic, or citric acid, was measured to assess the possibility of applying them also on vertical surfaces. Despite being constituted by the same concentration of both polysaccharide and acid, the viscosity decreases significantly from CS-Ac (280 P) to CS-Ox (80 P) and CS-Cit (40 P), indicating that the aggregation between the chitosan chains strongly depends on the number of carboxyl groups, as also evident from their photo in Figure S1.

The FTIR analysis was carried out to investigate the nature of these hydrogels. Therefore, to avoid the superimposition of signals of both solvent and plasticizer, the gels were prepared without glycerol and then dried at room temperature. The spectra, shown in Figures S2–S4, revealed that only acid–base reaction occurs without the formation of covalent bonds between the organic acids and chitosan, indicating that physical hydrogels are formed [43].

### 2.1. Cleaning of Lithotypes Stained with Rust Dispersion

#### 2.1.1. Photos and Colorimetry

**Figure 1:**
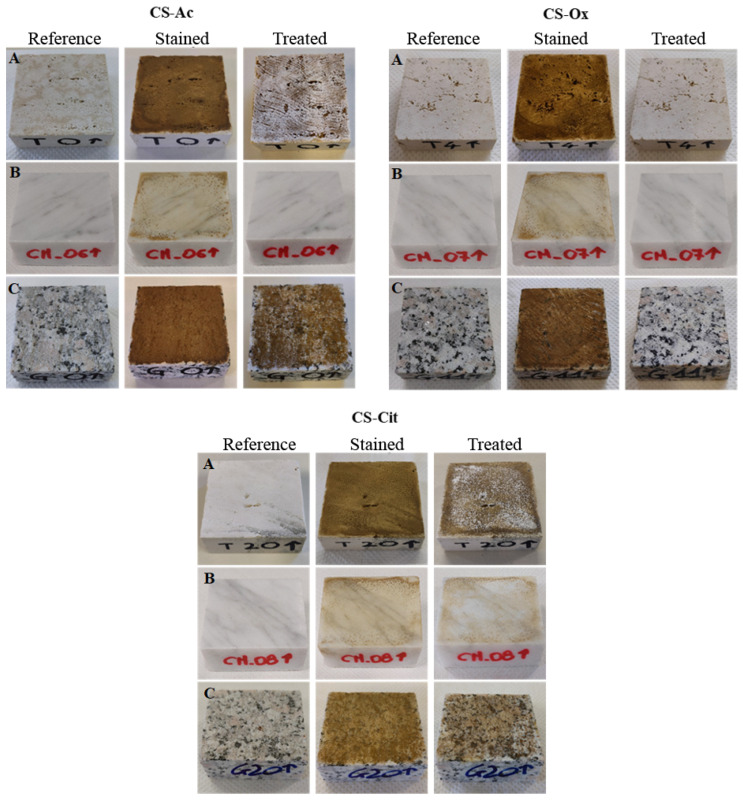
Photographs of the reference, stained (with a rust dispersion) and treated with Cs-Ac, CS-Ox, and CS-Cit specimens; (**A**) travertine, (**B**) marble, and (**C**) granite.

Three samples of granite, travertine, and marble stained with a rust dispersion were treated with the hydrogels, and their effectiveness in removing the iron stains was evaluated by photographing the surface of the samples during the cleaning procedure. Figure 1 reports, for each CS-acid and lithotype, the images of one of the three specimens before the staining (reference), soon after the rust deposition (stained), and after the treatment (treated).

It is evident from the figure that the degree of staining depends on the nature of the lithotype. Marble is less susceptible to stains than travertine and granite due to its polished surface and low water absorption.

After the treatment, CS-Ox effectively removes rust products from all the samples, whereas with CS-Cit achieves only a partial, unsatisfying, cleaning. Differently, Cs-Ac appears effective on marble specimen, but leaves visible residues on travertine and granite surfaces.

After a cleaning intervention, colorimetric analysis allows a deeper evaluation of the restoration of the original chromaticity and the gloss of a substrate. It is reported that color variations ΔE* < 3 result imperceptible to the human eye, while any treatment showing ΔE* < 5 is considered acceptable for conservative purposes [44]. Table 1 lists the mean colorimetric parameters of the reference sample, L*, a*, and b*, their variations after staining and treatment, as well as their corresponding ΔE* values, calculated according to Equation (1) (Section 4.2.3).

In stained granite and travertine samples, the global chromatic differences show values ΔE* ≈ 35, while marble specimens exhibit ΔE* ≈ 17, a value halved compared to the other two stones. Whatever the extent of the chromatic variation on each specimen, it is mainly due to the darkening, reddening, and yellowing of the surfaces as highlighted by the signs of the values of ΔL*, Δa*, and Δb*.

After the treatment with CS-Cit, the colorimetric coordinate values for all materials decrease, but the resulting chromatic variation turns out to always be higher than 10, a value not acceptable for conservative purposes. Similar results are observed following the treatment with the hydrogel containing acetic acid, with the sole exception of the marble specimens. By applying CS-Ac, the granite remains almost unaffected, and the travertine results only partially cleaned. On the contrary, the chromaticity of marble results is completely restored after only one treatment, with the recovery of all the colorimetric coordinates that provide accordingly ΔE* = 1, a value well below the perception limit of the human eye. Excellent results are obtained using the formulation containing the oxalic acid; indeed, a single treatment results enough to effectively restore the original chroma of the substrates regardless of the nature of the lithotype. As highlighted in Table 1, all the colorimetric coordinates return to the original reference values with a consequent negligible ΔE*.

**Scheme 1:**
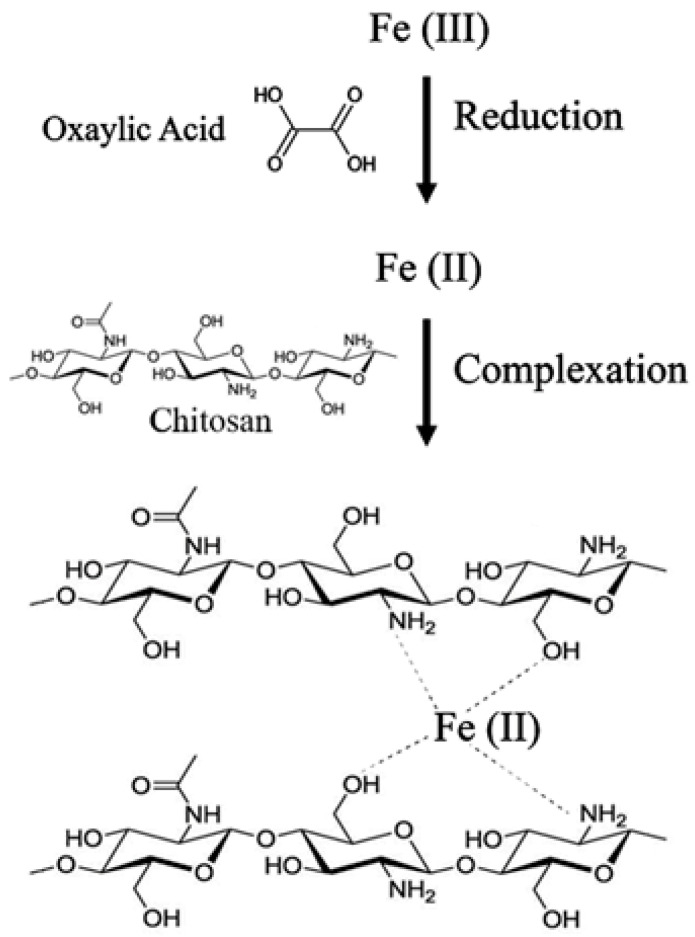
Proposed mechanism of action of the chitosan-oxalic acid hydrogel in reducing Fe(III) and subsequent chelation of Fe(II) by chitosan.

Recently, it has been reported that 1.6 M aqueous solution of oxalic acid, applied for 24 h in a cellulose poultice, is capable of effectively removing iron crusts from sandstone [14]. In this work we report similar results, but with an acid concentration six times lower, i.e., 0.25 M. Scheme 1 reports the hypothesized mechanism, in which the reducing ability of oxalic acid is combined with the chelating properties of chitosan.

As evident from the scheme, the complexation of Fe(II) involves both the amino and the C6-hydroxyl groups of chitosan, according to a previous study [45]. Therefore, the greater effectiveness of the CS-Ox hydrogel, compared to the poultice, could be attributed to the better ability of chitosan to chelate iron ions.

Based on these results, the CS-Ox hydrogel was selected to investigate its efficacy also at a microscopic level.

#### 2.1.2. ^1^H-NMR Relaxometry

Relaxometry is a powerful technique for studying water confined in porous materials [46,47,48,49]. In fact, according to NMR theory, when the sample is fully water-saturated, there exists a correlation between the inverse Laplace transform of the proton T_2_-decay of water and the pore-size distribution of the material [50].

In our experiment, the transverse relaxation decays of water-saturated reference, stained, and treated stone samples were acquired and directly compared, avoiding the inverse Laplace transform. Moreover, as the T_2_-decays were recorded under stationary conditions of water absorption, there are no effects due to the time of water absorption are possible. Unfortunately, due to the presence of a high amount of intrinsic paramagnetic species in granite and the very low porosity of marble, it was only possible to apply this technique to travertine.

**Figure 2:**
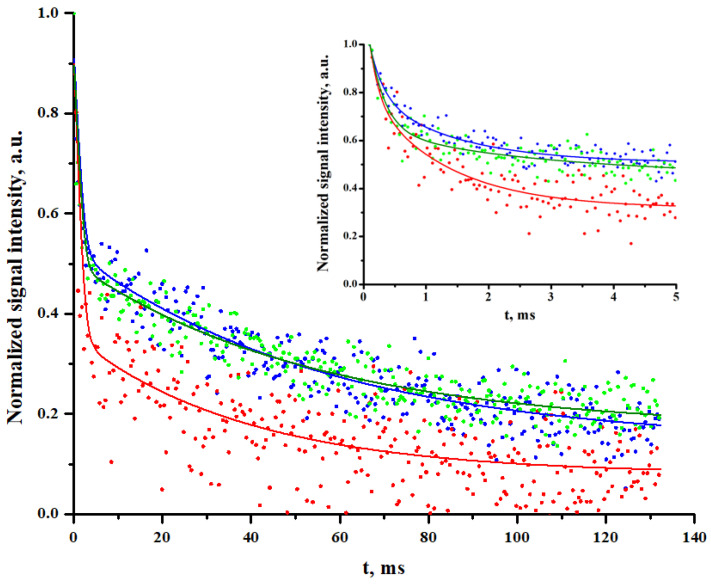
Water T_2_ decays by NMR relaxometry on travertine for reference (blue), stained with rust dispersion (red), and treated with CS-Ox (green) samples. In the inset, the curves are plotted in the first 5 ms of decay.

The travertine, which is mainly composed of calcium carbonate and characterized by large cavities, was successfully analyzed by ^1^H-NMR T_2_ relaxometry and the normalyzed signal decays of reference, stained and cleaned with CS-Ox samples are compared in Figure 2.

The red curve, corresponding to the stained samples, shows a faster decay than that of the reference (blue curve), suggesting the presence of rust products on the stone surface, which affects the relaxation process of the water molecules. In fact, being constant the surface-to-volume ratio of the sample as well as the echo time, the faster relaxation time of water can be imputed to the paramagnetic nuclei deposited within the porous structure. In fact, the presence of a few ppm of paramagnetic impurities, such as Fe(III) ions, can significantly modify the transverse relaxation times of confined water molecules. These impurities act as relaxation centers for protons, causing a reduction in the magnitude of the relaxation processes. In fact, their unpaired electrons generate local magnetic fields that interact with the nuclear spins of nearby water molecules, leading to a faster T_2_-decay [46,48,51].

After the cleaning procedure with CS-Ox, the decay signal (green curve) appears restored and overlaps almost completely with the reference signal. The differences highlighted here could be better appreciated in the inset of Figure 2, in which the decay signals in the first 5 ms are shown.

The experimental evidence of the T_2_ decays suggest that iron products are completely removed from the stone surface, confirming the above discussed macroscopic observations.

#### 2.1.3. SEM-EDS Analysis

**Figure 3:**
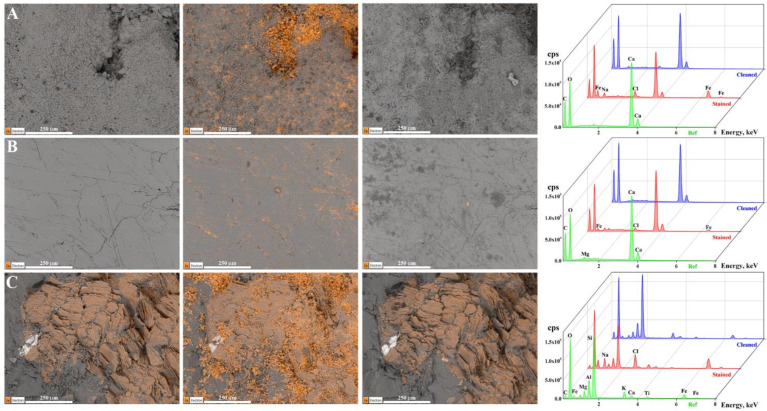
SEM-EDS analyses of 1 mm^2^ area of travertine (**A**), marble (**B**) and granite (**C**), in which the presence of Fe is highlighted in orange, before rust deposition (on the **left**)), after the staining (in the **middle**) and after the treatment with CS-Ox (on the **right**). In the last column, for each lithotype, the graphics 3D of EDS spectra in the three phases of the procedure.

All the lithotypes were observed through an electronic microscope to identify four areas of 1 mm^2^ on the stone samples. EDS microanalyses were performed on each of them before the deposition of rust suspension (reference), after the staining, and after the treatment with CS-Ox. For each lithotype, Figure 3 shows the SEM images, with iron highlighted in orange, of the reference, stained, and treated areas and the 3D comparison between the corresponding EDS spectra.

As expected, before the deposition of rust suspension, the two carbonate-based substrates (Figure 3A,B) do not exhibit appreciable iron content; on the contrary, several Fe-domains are almost ubiquitous in the granite sample (Figure 3C).

After the staining of the stone surfaces, a significant presence of iron is observed also on travertine and marble, as evidenced by the presence of orange spots covering the investigated areas and semi-quantified by their corresponding EDS spectra. Marble is characterized by the lowest content of deposited ferrous species, which is in full agreement with its morphology and macroscopic analysis.

On all stained samples, the presence of Na and Cl are also detected, which is due to the sodium chloride, added for accelerating the iron oxidation process.

After the treatment with the CS-Ox, the deposited iron appears completely removed from the surface of the three specimens, as evidenced by the lack of the orange spots on SEM images and the near overlap of the EDS spectra of the treated and reference samples. In addition, the absence of nitrogen, detectable at 0.39 KeV, sodium and chlorine in the EDS spectra of the treated samples, indicates the complete removal of the hydrogel that, evidently, is able to adsorb also the sodium chloride deposited during the staining process.

To better interpret the results, the weight percentages of the most characterizing elements for each lithotype, as well as of iron, were evaluated on four selected areas of all the specimens, in the three phases of the procedure (Table 2).

Reference samples of travertine and marble show high reproducibility in their main constituting element, i.e., carbon and calcium, according to their carbonate nature. Differently, due to its coarse and multicomponent nature, granite shows different distributions of silicon and aluminum depending on the investigated area. Moreover, it is evident that the reference sample of travertine presents only traces of iron (0.1% wt), while for all the investigated areas of marble, the metal is not present at all. In contrast, the iron content of the different observed areas of granite ranges between 0.6 and 12%, showing an inhomogeneous distribution of ferrous compounds in the material.

Although the Fe content of all samples increases significantly after the deposition of rust, marble appears to be the less contaminated specimen with an overall iron abundance of only 3%, compared to travertine and granite, whose amounts are over 10%. Following the treatment, the ‘natural’ amount of iron of the pristine stone substrates is almost completely restored.

SEM-EDS analysis confirms not only the good ability of CS-Ox in removing iron stains of all stones, but also that the hydrogel is capable of removing other hygroscopic contaminants from the surface of the substrates without significantly affecting their morphology.

### 2.2. Cleaning of Lithotypes Stained with Iron Grid

#### 2.2.1. Photos and Colorimetry

**Figure 4:**
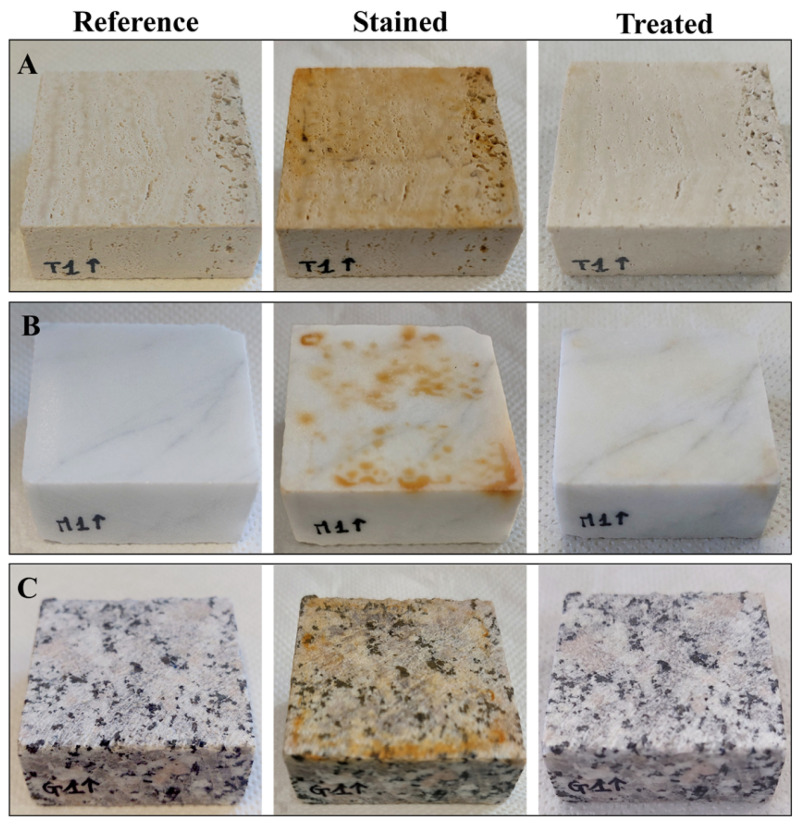
Photographs of the reference, stained with rusty iron grid and treated specimens subjected to the treatment with CS-Ox; (**A**) travertine, (**B**) marble, and (**C**) granite.

To more faithfully reproduce the staining process that stones undergo when in contact with iron elements, three specimens for each lithotype were artificially polluted by placing a rusty iron grid in contact with one of their larger surfaces. For each lithotype, Figure 4 shows the photos of one of the three samples before the staining (reference), after the staining and following the treatment with CS-Ox.

The marble sample exhibits a spotted deposition of rust on its surface, in contrast to travertine and granite, whose surfaces are more homogeneously stained. Nevertheless, one treatment is sufficient to remove the rust formed on the travertine and granite samples, while a residual light orange color is still perceptible on the marble surface.

The colorimetric analysis was then performed to evaluate the changes in the substrate color and Table 3 reports the mean values of chromatic coordinates of each reference stone, their difference in the stained and treated surfaces with their respective ΔE* values.

Although the color alteration of the samples stained with the iron grid results is slightly lower than that obtained with the deposition of the rust dispersion (Table 1), all stones result darker and yellower than the reference. The colorimetric coordinates of granite, after a single treatment with CS-Ox, appear to be similar to the reference samples, and the ΔE* values turn out to be below the perception limit of the human eye. Although the stains on travertine samples appear completely removed from the treated surface (see Figure 4A), colorimetric analysis reveals a slightly perceptible color alteration, comparable to that of marble specimens. In fact, for both carbonate stones, the ΔE* is equal to 3, and it is mainly attributable to the positive shift of the b* coordinate, indicating a partial yellowing of their surfaces compared to the pristine material.

Colorimetric and photographic analyses have shown that a single treatment with CS-Ox is sufficient to completely restore the original color of the granite surface and to reduce the color change caused by rust stains on travertine and marble to values acceptable for restoration purposes, less than 5.

#### 2.2.2. Stereomicroscopy

**Figure 5:**
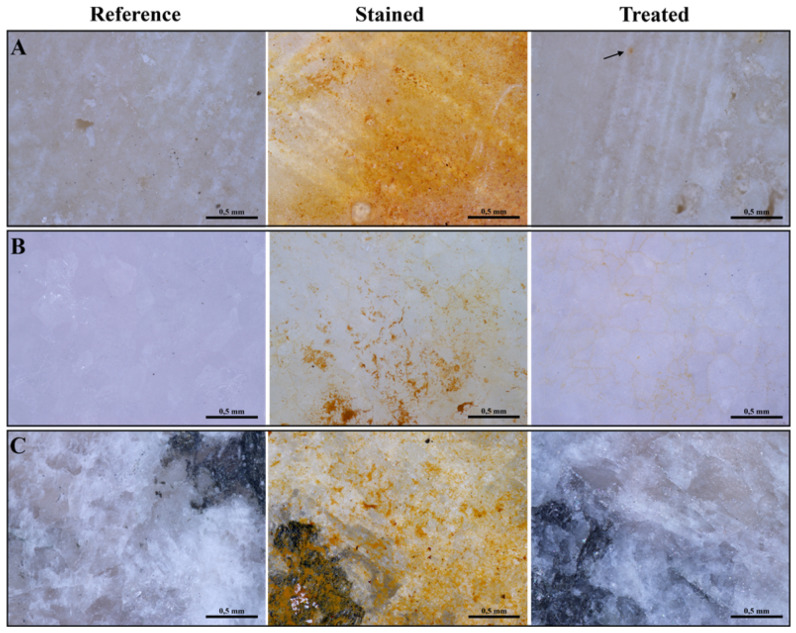
Stereomicroscope images at 56× of magnification of travertine (**A**), marble (**B**), and granite (**C**), reference, stained and treated with CS-Ox surfaces. In panel A (right), an evident spot of rust on the travertine-treated surface is indicated by a black arrow. Bar = 0.5 mm.

Microscopic observations were made on the treated surface of all samples in order to study the residual staining of carbonate stones and to confirm the high effectiveness of the treatment on granite. For each lithotype, Figure 5 reports micrographs of the reference, stained and treated surfaces at 56× of magnification.

The micrographs of the stained samples reflect the evidence highlighted by photography and colorimetry. In fact, on travertine and granite, a homogeneous deposition of rust can be observed, while on marble, the less stained stone, a thinner and more spotted layer can be seen.

The travertine surface gives results almost entirely comparable to the reference, except for a few small rust spots, approximately 50 µm in size, one of which is indicated by the arrow in Figure 5A. Differently, on marble surface (Figure 5B), the rust residues are confined between the boundary areas of the interlocking grains of calcite. This evidence can explain the slight coloration revealed by the colorimetric analysis, which is also partially visible to the naked eye. Anyway, these residues are strongly bound to the marble surface and are the most challenging to remove, as also already reported in the literature [5,20]. Therefore, further studies will be necessary to find new formulations capable of completely cleaning marble stone or, alternately, to develop protective agents capable of avoiding the deposition of such residues.

Regarding granite, the microscope analysis (Figure 5C) confirms the absence of rust residues on the treated surface and the complete restoration of its original features, as previously obtained when the lithotype was stained with the dispersion of corrosion products.

Comparing the results of carbonate stones, CS-Ox hydrogel is able to clean both stones stained with the rust dispersion better than those stained with the iron grids, despite they resulted more contaminated (see ΔE* values in Table 1 and Table 3). This phenomenon could be attributed to the different sizes of the rust particles involved in the staining processes. In fact, when the iron grid is immersed in an aqueous salt solution, nanoparticles of corrosion products are formed, which then interact to form large aggregates. Thus, the rust dispersion used for the first experiments should consist of large aggregates that can effectively adhere to the sample surface but are unable to penetrate into the material. Differently, the rusted iron grid could transfer nanometer-sized particles, which spread more easily and deeper into the stone [9].

## 3. Conclusions

Both the stability and low solubility of ferrous species make rust removal from monumental stone surfaces a crucial issue. Chitosan-carboxylic acid hydrogels, optimized by combining the reducing ability of carboxylic acids with the intrinsic chelating properties of the polysaccharide, have been applied on granite, travertine and marble samples, artificially stained with rust dispersion. Photos and colorimetric parameters have shown that the formulation containing oxalic acid is the most effective in removing rust stains from all the specimens with just one treatment. The good cleaning performance of CS-Ox has also been confirmed by NMR and SEM/EDS analysis, which highlighted the ability of the hydrogel to remove iron and undesired hygroscopic pollutants from the stone surface. Moreover, similar results have been achieved on samples stained by placing a rusty iron grid on their surfaces to more faithfully reproduce a natural staining process. Colorimetry has highlighted that, despite the color difference between the treated and reference samples being slightly higher than those artificially stained with rust dispersion, they are well below the acceptable limit for a restoration intervention. In particular, small rust residues on the surfaces of travertine and between the grains of marble have been evidenced by the stereomicroscope observations.

The here optimized cleaning protocol will be tested “in situ” on naturally stained artworks. Nevertheless, further studies will be aimed at finding other formulations that can be applied to marble and travertine to completely remove iron residues. In addition, strategies aimed at protecting stone surfaces in contact with iron elements will be investigated to prevent the deposition of rust and to facilitate its removal.

## 4. Materials and Methods

### 4.1. Materials

Three lithotypes were selected for this study among the most used stone materials in built heritage i.e., granite, travertine and marble. Cut specimens of approximately 5 × 5 × 2 cm^3^ were purchased from Elia Marmi s.n.c. L’Aquila (AQ), Italy.

*Sardinian pink granite* is a medium-grained (0.5–2 cm) material with an open porosity that ranges from 0.5 to 1%. It is mainly composed of silicates and aluminosilicates, such as plagioclase (35%), quartz (31%), which confers the typical shine of the material, K-feldspar (24%), responsible of the pinkish inclusions, and biotite (10%) [52,53].

*Roman travertine* is a calcareous sedimentary rock mainly composed of CaCO_3_ (97–99%) and appears as a white-beige stone characterized by 5 to 15% open porosity, mostly due to the presence of cavities and macropores [54,55].

*Carrara marble* is a metamorphic stone, with a grain size ranging from 0.12 to 0.35 mm and a very low open porosity of about 0.5% [56]. It is primarily composed of calcium carbonate with low content of magnesium carbonate and other trace elements. White Carrara marble, also called “Ordinary white”, the widely diffused and commercially available, appears as a pearl-white stone with typical grey veins and spots [57].

Low viscosity chitosan with 77% deacetylation degree and 590 kDa average molecular weight [58], acetic acid, oxalic acid dihydrate, citric acid monohydrate and glycerol were supplied by Sigma-Aldrich (St. Louise, MO, USA) and were used as received.

### 4.2. Methods

#### 4.2.1. Staining of Stone Surfaces

Iron grids were immersed in a solution containing sodium chloride (≈1 M) to accelerate the oxidation process and obtain a dispersion of corrosion products. To reach a reproducible staining of the lithic substrates, regardless of their nature, 1 mL of the rust dispersion, containing about 28 mg of the oxidized compounds, was deposited on one of the 5 × 5 cm^2^ surfaces of three specimens for each lithotype.

The other three samples for lithotype were stained by placing the rusted grids directly on one of their surfaces to simulate a more realistic pollution. All stone specimens were kept submerged in distilled water for about a week to favor the deposition of corrosion products on their surfaces.

#### 4.2.2. Hydrogel Preparation

Three chitosan-based hydrogels containing acetic (CS-Ac), oxalic (CS-Ox), and citric (CS-Cit) acids were prepared.

Chitosan (5% *w*/*w*) was dissolved in an aqueous solution acidified with the minimum amount of carboxylic acid capable of completely dispersing the polysaccharide, i.e., 1.5%, 3% and 4% *w*/*w* of acetic, oxalic, or citric acid, respectively, all corresponding to about 0.25 M. The viscosity of the hydrogels was measured at room temperature using a Fungilab Viscolead mod. ADV L rotational viscometer (Fungilab, Barcelona, Spain) (spindle L4 at 20 rpm).

To peel off the dried gels more easily and avoid several rinses, 3% *w*/*w* of glycerol, which is commonly used as a plasticizer, was added to all the formulations. The hydrogels were then applied to the stained stone surfaces with the aid of cotton gauze to facilitate their removal after approximately 24 h, the time required for the gel to dry at room temperature.

#### 4.2.3. Colorimetry

Chromatic changes of both stained and treated surfaces were assessed by colorimetric analysis and compared to data before staining. The measurements were performed by means of a Sama Tools SA230 (Sama Tools, Viareggio, LU, Italy) portable colorimeter working in SCE mode with an 8° standard angle observer, light D65, and temperature of 6504 K (average daylight, including the UV region). For all specimens, 25 points were acquired by using a 5 × 5 grid to cover approximately 60% of the total area. The colorimetric coordinates, L*, a*, and b*, were acquired in the CIELAB color space proposed in 1976 by the International Lighting Commission (CIE) [59]. Color differences, expressed in terms of ΔL*, Δa*, and Δb*, were calculated between both the stained and treated surfaces and their reference (surface before staining). By means of a vector sum, the color alteration values, expressed as ΔE*, were calculated using Equation (1):

(1) $$\Delta E^{*}=\sqrt{\Delta L^{*2}+\Delta a^{*2}+\Delta b^{*2}}$$

#### 4.2.4. ^1^H-NMR Relaxometry

Non-destructive relaxometry was performed using the NMR equipment mq-Profiler (Bruker, Milan, Italy), which consisted of a surface probe with a portable electronic apparatus. The coil in use works at a Larmor frequency of 17.8 MHz, can be put in contact with the sample surface of whatever dimension and can excite water protons up to 2 mm deep from its surface with a sensitive volume of 2 × 0.2 × 0.8 cm^3^ (x, y, z). Fully water-saturated conditions for the stones were obtained by placing the air-dried samples under a vacuum for 30 min and then keeping them submerged in distilled water for another 30 min, after which they were wrapped in polyethylene foils to avoid water evaporation during the NMR measurement. The T_2_ signal decays were acquired by means of a CPMG pulse sequence of 3000 echoes with the shortest possible echo time of 44 μs to reduce the diffusion effect. In addition, 512 scans were performed by repeating the sequence every 2 ss to improve the signal/noise ratio. Signal decays of water absorbed in stained and treated samples were compared with that of the reference.

#### 4.2.5. Microscopy Analysis

SEM-EDS analyses or stereomicroscopy were performed to evaluate, at a microscopic level, the efficacy of hydrogels in removing iron oxides from the surface of lithotypes. For SEM/EDS measurements, a Zeiss GeminiSEM 500 (Zeiss, Jena, Germany) equipped with EDS OXFORD Aztec Energy with INCA X-ACT detector was used; all measures were carried out in variable pressure mode (VP) with an accelerating voltage of 15 keV and a working distance of 8.5 mm. Four areas of about 1 mm^2^ of each stone were selected, and their position was saved to correlate the elemental composition changes during the experimental procedure. Therefore, EDS microanalysis was performed on all the investigated areas before, soon after the staining process, and after the cleaning treatment.

Micrographs were acquired by using an AxioZoom V16 (Zeiss, Jena, Germany) stereomicroscope at a 56× magnification. The images were then elaborated by means of the Zen Blue 3.3 software.

## Figures and Tables

**Table 1:** For each lithotype subjected to the treatment with CS-Ac, CS-Ox, or CS-Cit, the mean chromatic coordinates of the reference stone, mean chromatic coordinates differences of both stained (with a rust dispersion) and treated specimens, and the corresponding mean color differences.

**CS-Ac**	**Travertine**		L*	a*	b*	
		**Ref**	77 ± 2	3.9 ± 0.4	10 ± 1	
			ΔL*	Δa*	Δb*	ΔE*
		**Stained**	−33 ± 3	5 ± 1	16 ± 2	**37**
		**Treated**	−11 ± 2	1.1 ± 0.5	2 ± 1	**11**
	**Marble**		L*	a*	b*	
		**Ref**	76 ± 2	−1.4 ± 0.1	−2.1 ± 0.3	
			ΔL*	Δa*	Δb*	ΔE*
		**Stained**	−9 ± 3	2.4 ± 0.9	11 ± 2	**15**
		**Treated**	−1 ± 2	0.1 ± 0.1	0.4 ± 0.4	**1**
	**Granite**		L*	a*	b*	
		**Ref**	65 ± 3	0.6 ± 0.6	2.4± 0.9	
			ΔL*	Δa*	Δb*	ΔE*
		**Stained**	−23 ± 3	8.2 ± 0.8	23 ± 1	**34**
		**Treated**	−19 ± 2	5.3 ± 0.7	14 ± 2	**24**
**CS-Ox**	**Travertine**		L*	a*	b*	
		**Ref**	77 ± 1	3.3 ± 0.2	7.5 ± 0.5	
			ΔL*	Δa*	Δb*	ΔE*
		**Stained**	−27 ± 3	7.3 ± 0.6	21 ± 1	**35**
		**Treated**	−1 ± 1	−0.2 ± 0.3	0.6 ± 0.7	**1**
	**Marble**		L*	a*	b*	
		**Ref**	75 ± 2	−1.3 ± 0.2	−1.7 ± 0.3	
			ΔL*	Δa*	Δb*	ΔE*
		**Stained**	−11 ± 4	3 ± 1	13 ± 2	**17**
		**Treated**	−1 ± 2	0.0 ± 0.2	0.5 ± 0.4	**1**
	**Granite**		L*	a*	b*	
		**Ref**	69 ± 3	0.2 ± 0.8	1 ± 1	
			ΔL*	Δa*	Δb*	ΔE*
		**Stained**	−25 ± 3	8 ± 1	22 ± 2	**34**
		**Treated**	−1 ± 2	0.1 ± 0.6	0.1 ± 0.7	**1**
**CS-Cit**	**Travertine**		L*	a*	b*	
		**Ref**	81 ± 3	3.1 ± 0.3	8 ± 1	
			ΔL*	Δa*	Δb*	ΔE*
		**Stained**	−34 ± 3	6.7 ± 0.6	19 ± 1	**40**
		**Treated**	−15 ± 6	2 ± 1	4 ± 2	**16**
	**Marble**		L*	a*	b*	
		**Ref**	74 ± 3	−1.4 ± 0.2	−1.9 ± 0.3	
			ΔL*	Δa*	Δb*	ΔE*
		**Stained**	−11 ± 3	3 ± 1	13 ± 2	**17**
		**Treated**	−4 ± 4	2 ± 1	9 ± 3	**10**
	**Granite**		L*	a*	b*	
		**Ref**	67 ± 4	0.3 ± 0.8	2 ± 1	
			ΔL*	Δa*	Δb*	ΔE*
		**Stained**	−17 ± 2	6.2 ± 0.7	18 ± 2	**26**
		**Treated**	−11 ± 3	2.5 ± 0.9	7± 2	**13**

**Table 2:** Weight percentages of most abundant constituents, including Fe, of travertine, marble, and granite, determined in four areas of each reference, stained with rust and cleaned sample and the corresponding mean values.

**Travertine**						
	**Element**	**Area 1**	**Area 2**	**Area 3**	**Area 4**	**Mean**
**Reference**	Ca	40.2	40.1	39.0	40.1	39.9 ± 0.6
	C	13.7	13.7	14.3	13.9	13.9 ± 0.3
	Fe	0.1	0.1	0.1	0.1	0.1 ± 0.0
**Stained**	Ca	32.3	30.5	26.3	30.8	30 ± 3
	C	13.2	13.5	14.0	16.8	14 ± 2
	Fe	9.0	11.1	15.9	6.2	11 ± 4
**Treated**	Ca	34.8	36.9	33.1	35.5	35 ± 2
	C	16.7	15.3	17.2	15.8	16.3 ± 0.9
	Fe	0.1	0.2	0.2	0.2	0.2 ± 0.1
**Marble**						
	**Element**	**Area 1**	**Area 2**	**Area 3**	**Area 4**	**Mean**
**Reference**	Ca	37.2	38.3	37.6	38.0	37.8 ± 0.5
	C	16.2	15.0	15.8	15.3	15.6 ± 0.5
	Fe	0.0	0.0	0.0	0.0	0.0
**Stained**	Ca	36.9	35.9	36.1	37.0	36.5 ± 0.6
	C	14.4	13.9	13.9	13.5	13.9 ± 0.4
	Fe	2.0	3.6	2.9	2.4	2.7 ± 0.7
**Treated**	Ca	31.5	32.6	32.3	32.8	32.3 ± 0.6
	C	17.5	16.7	16.5	16.8	16.9 ± 0.4
	Fe	0.1	0.2	0.2	0.4	0.2 ± 0.1
**Granite**						
	**Element**	**Area 1**	**Area 2**	**Area 3**	**Area 4**	**Mean**
**Reference**	Si	32.4	23.5	26.6	30.8	28 ± 4
	Al	5.3	6.0	9.7	3.0	6 ± 3
	Fe	0.9	12.0	0.6	6.7	5 ± 5
**Stained**	Si	24.8	14.3	17.5	24.7	20 ± 5
	Al	4.3	3.2	6.4	2.3	4 ± 2
	Fe	14.0	27.3	16.0	15.3	18 ± 6
**Treated**	Si	31.5	21.6	24.8	28.5	27 ± 4
	Al	4.9	4.6	9.2	2.2	5 ± 3
	Fe	0.8	10.2	0.5	7.1	5 ± 5

**Table 3:** For each lithotype subjected to the treatment with CS-Ox, the mean chromatic coordinates of the reference stone, mean chromatic coordinate differences of both stained (with rusty iron grid) and cleaned specimens, and the corresponding mean color differences.

**Travertine**		L*	a*	b*	
	**Ref**	78 ± 4	3.6 ± 0.3	9 ± 1	
		ΔL*	Δa*	Δb*	ΔE*
	**Stained**	−7 ± 3	6 ± 3	16 ± 5	**19**
	**Treated**	0 ± 2	0.8 ± 0.4	2 ± 1	**3**
**Marble**		L*	a*	b*	
	**Ref**	79 ± 4	−0.8 ± 0.2	−2.1 ± 0.7	
		ΔL*	Δa*	Δb*	ΔE*
	**Stained**	−5 ± 4	3 ± 2	11 ± 5	**13**
	**Treated**	0 ± 1	0.4 ± 0.4	3 ± 2	**3**
**Granite**		L*	a*	b*	
	**Ref**	70 ± 5	−0.2 ± 0.8	1 ± 2	
		ΔL*	Δa*	Δb*	ΔE*
	**Stained**	−11 ± 9	6 ± 3	16 ± 6	**21**
	**Treated**	−1 ± 2	−0.1 ± 0.6	−0.3 ± 0.6	**2**

## Data Availability

The data presented in this study are openly available in the article.

## Supplementary Materials

The following supporting information can be downloaded at https://www.mdpi.com/article/10.3390/gels10060359/s1, Figure S1: Photograph of the physical hydrogels Cs-Ac, CS-Ox and CS-Cit; Figure S2: FTIR spectra of acetic acid (red), pure chitosan (black) and CS-Ac dried hydrogel (blue); Figure S3: FTIR spectra of oxalic acid (red), pure chitosan (black) and CS-Ox dried hydrogel (blue); Figure S4: FTIR spectra of citric acid (red), pure chitosan (black) and CS-Cit dried hydrogel (blue). References [43,58].
