# Supplementary material for: Efficacy of Chitosan-Carboxylic Acid Hydrogels in Reducing and Chelating Iron for the Removal of Rust from Stone Surface
Source: Gels. 2024 May 22;10(6):359. doi: 10.3390/gels10060359 (PMC11202951; doi:10.3390/gels10060359)
Supplement: Supplementary file 1 [file gels-10-00359-s001.zip › gels-2995113-supplementary.pdf]

Supplementary Materials

Figure S1 shows the photograph of the physical hydrogels CS-Ac, CS-Ox and CS-Cit.

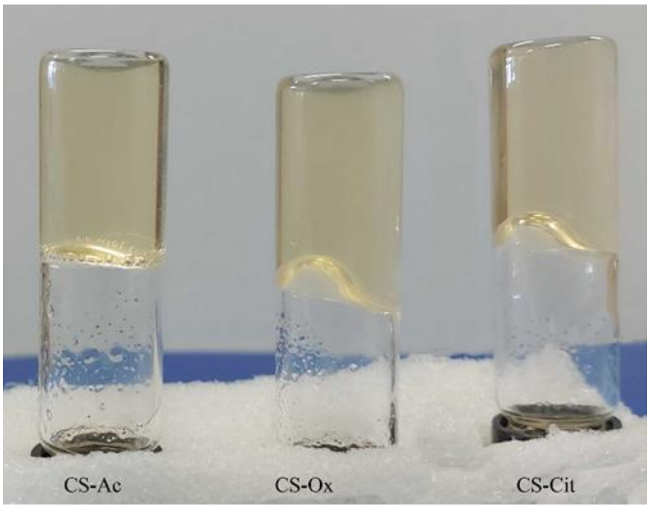

Figure S1. Photograph of the physical hydrogels Cs-Ac, CS-Ox and CS-Cit.

FTIR-ATR analysis

FTIR spectra were acquired carrying out 8 scans at a resolution of 4 cm^{-1} in the spectral range between 4000 cm^{-1} and 400 $cm^{-1}$by means of Perkin-Elmer SpectrumTwo FT-IR spectrophotometer equipped with the universal total attenuated reflectance tool (UATR).

The FTIR spectra of the dry gels, prepared without glycerol to avoid the superimposition of the signals of both the solvent and the plasticizer, along with that of pure chitosan and the acid are reported in Figure S2-S4.

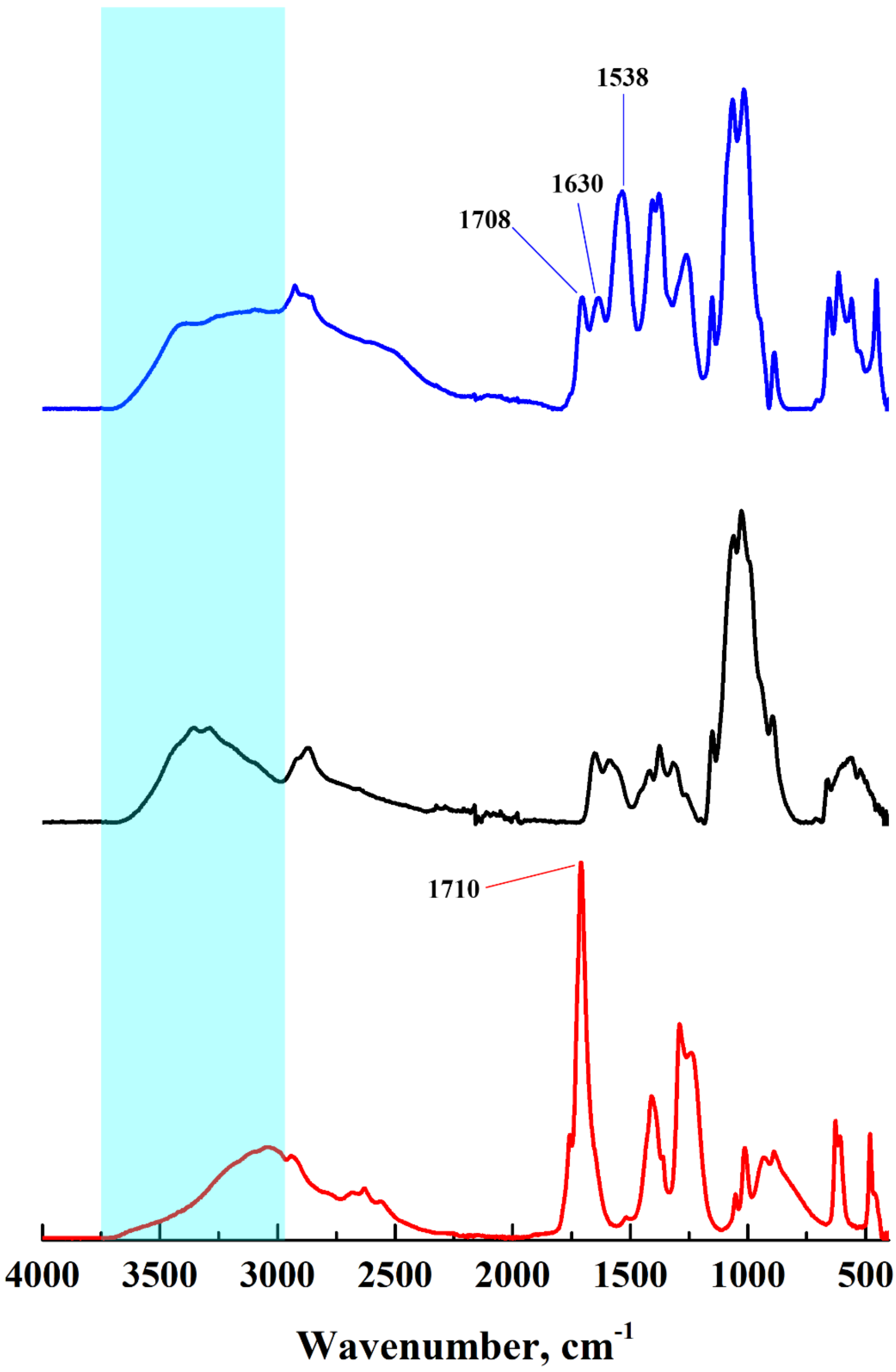

Figure S2. FTIR spectra of acetic acid (red), pure chitosan (black) and CS-Ac dried hydrogel (blue).

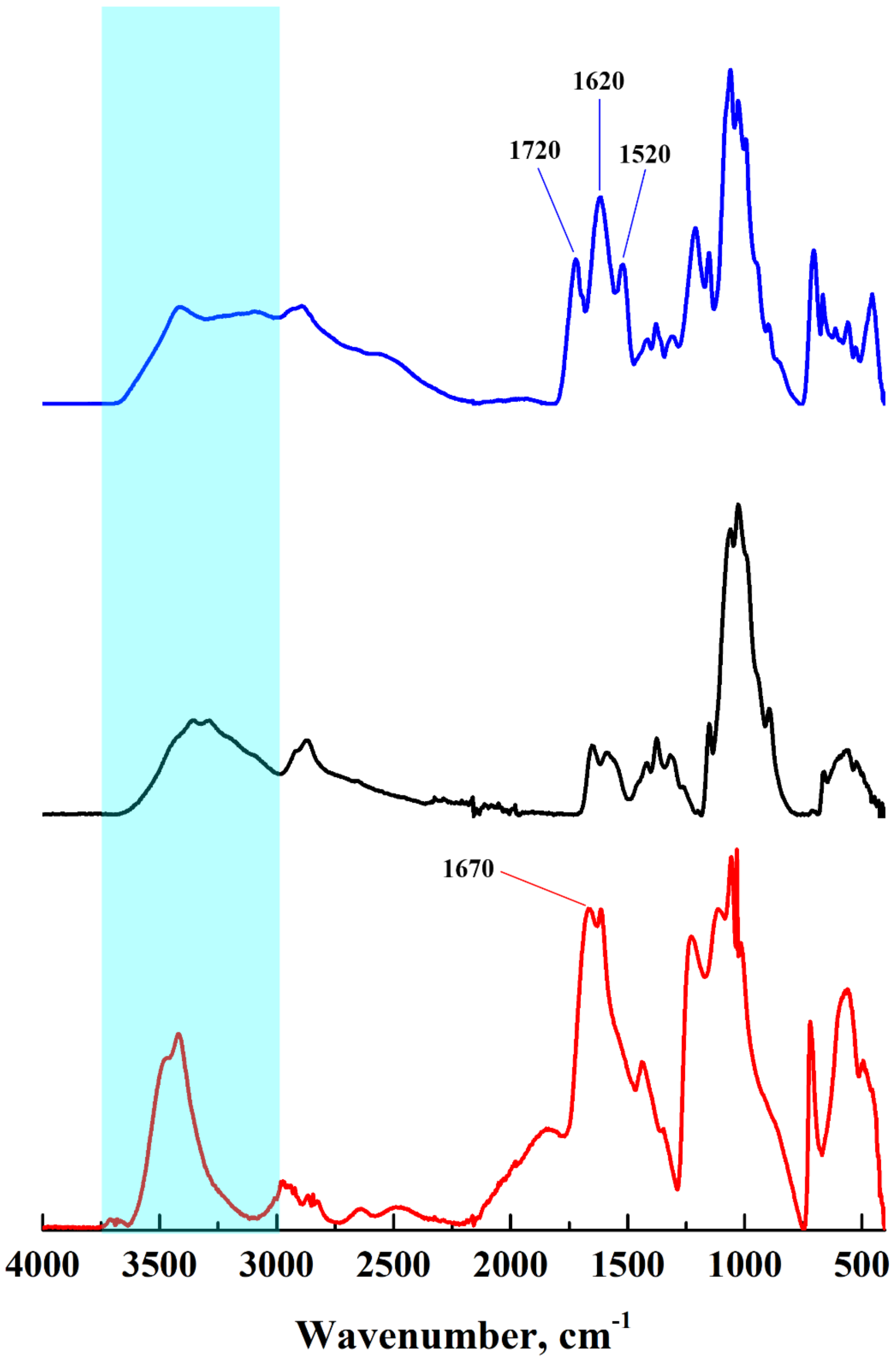

Figure S3. FTIR spectra of oxalic acid (red), pure chitosan (black) and CS-Ox dried hydrogel (blue).

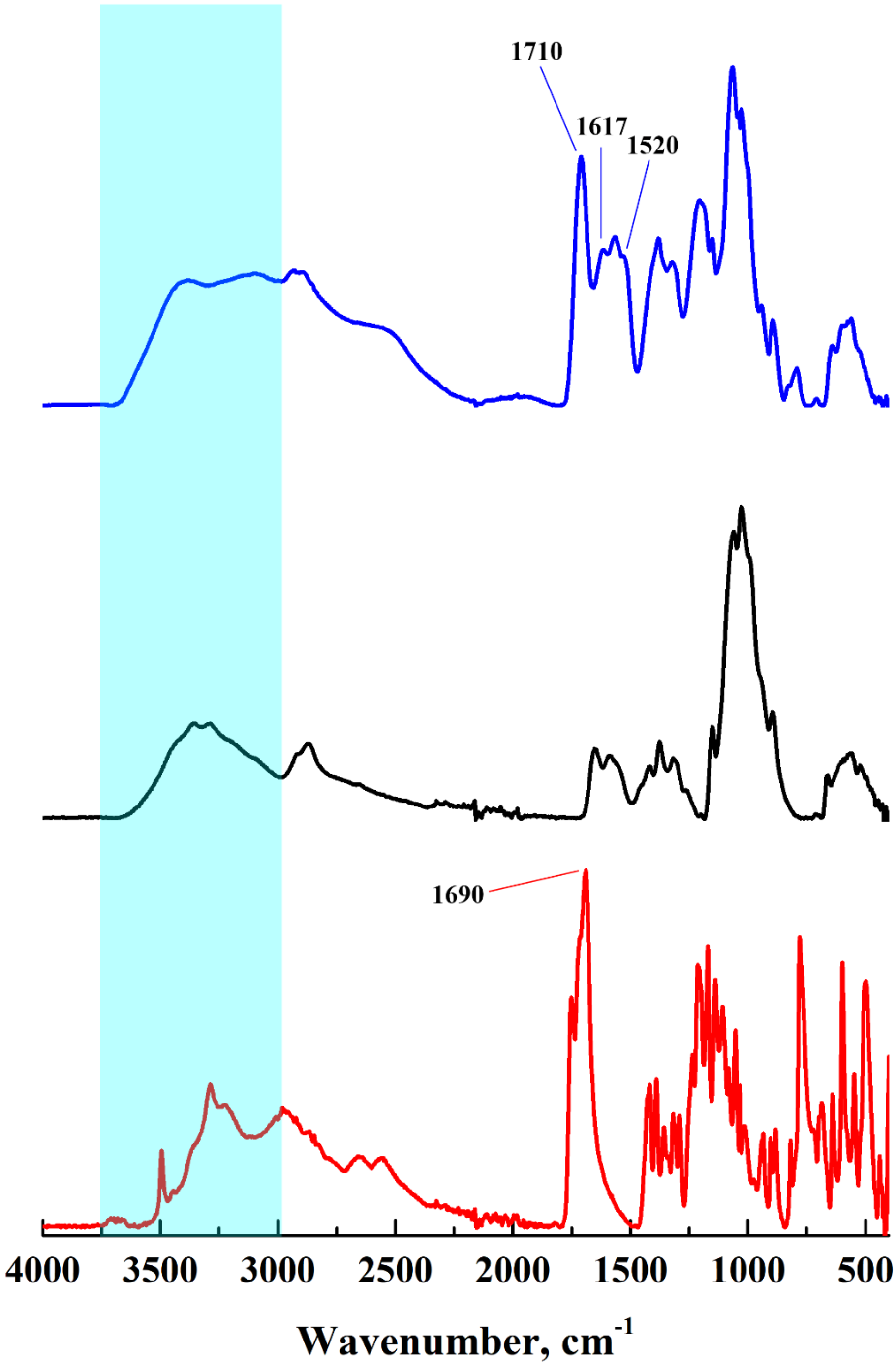

Figure S4. FTIR spectra of citric acid (red), pure chitosan (black) and CS-Cit dried hydrogel (blue).

Compared to pure chitosan (black curves in Figure S2, S3 and S4), the FTIR spectra of all dried gels (blue curves in Figure S2, S3 and S4) show a well-defined peak around 1715 cm^{-1}, corresponding to the stretching vibration of the carbonyl group of acetic, oxalic or citric acid. Carbonyl stretching of pure oxalic and citric acids results centered at slightly lower wavenumber due to their interactions with the crystallization water, which affect the vibrational mode of this functional group [43].

Part of the acid is deprotonated due to the acid-base reaction with chitosan, as evident from the absorption bands around 1625 cm^{-1}. In fact, in this region, the absorption bands of the asymmetric stretching of carbonyl function of carboxylates and the asymmetric bending of the ammonium ions are located; moreover, the presence of the absorption band of the symmetric vibrations of both functional groups is detectable (1521 cm^{-1}) [58].

Lastly, a broadening of the O-H and N-H stretching between 3750 and 3000 cm^{-1} can be observed in the FTIR spectra of the dried hydrogels. This is probably due to the contribution of the stretching vibration of the N-H bonds of the ammonium ions (generally visible at 3100 cm^{-1}) formed after the acid-base reaction between the components.

References

43. Bellamy L.J., Pace R.J. Hydrogen bonding in carboxylic acids—I. Oxalic acids *Spectrochim. Acta* **1963**, *19*, 435-442; DOI:10.1016/0371-1951(63)80055-7
58. Gabriele, F.; Donnadio, A.; Casciola, M.; Germani, R.; Spreti, N. Ionic and covalent crosslinking in chitosan-succinic acid membranes: effect on physicochemical properties. *Carbohydr. Polym.* **2021**, *251*, 117106; DOI:10.1016/j.carbpol.2020.117106
